# Improving maternal, neonatal and child health outcomes in low-resource settings: Translating research evidence to practice – report from The Third International Conference on Maternal, Newborn and Child Health

**DOI:** 10.1111/1471-0528.17609

**Published:** 2023-08-02

**Authors:** Robert L. Goldenberg, Shivaprasad S. Goudar, Avinash Kavi, Nancy F. Krebs, Richard J. Derman, Sarah Saleem, Elizabeth M. McClure

**Affiliations:** 1 Columbia University, New York, New York, USA; 2 KLE Academy of Higher Education and Research, Jawaharlal Nehru Medical College, Belagavi, India; 3 University of Colorado School of Medicine, Denver, Colorado, USA; 4 Thomas Jefferson University, Philadelphia, Pennsylvania, USA; 5 Aga Khan University, Karachi, Pakistan; 6 RTI International, Durham, North Carolina, USA

## 1 | INTRODUCTION

The articles in this supplement reflect presentations made during the third International Conference on Maternal, Newborn and Child Health: Translating Research Evidence to Practice. The conference took place at the Jawaharlal Nehru Medical College (JNMC) in Belagavi, India, between 11 and 13 November 2022, and was organised by the JNMC Women’s and Children’s Health Research Unit (JNMC Research Unit) of the KLE Academy of Higher Education and Research (deemed to be a university). The presentations, made by researchers, funders and policymakers from India and around the world, each reflected the research undertaken by the JNMC Research Unit focusing on women’s and children’s health in low- and middle-income countries (LMICs). This scientific meeting, which brought together stakeholders from various disciplines, was not limited to continuing and completed protocols, but also engaged stakeholders to initiate a dialogue to frame an agenda to translate research evidence into policy and practice.

## 2 | ARTICLES IN THIS SUPPLEMENT

This supplement contains 20 articles that reflect the wide range of research undertaken by the JNMC Research Unit, as well as the support of the many international funders, including the World Health Organization (WHO, Geneva, Switzerland), the US National Institutes of Health (NIH) (particularly the *Eunice Kennedy Shriver* National Institute of Child Health and Human Development (NICHD), Bethesda, MD, USA), the NICHD Global Network for Women’s and Children’s Health Research (Global Network), the Bill and Melinda Gates Foundation (BMGF) (Seattle, WA, USA), the Children Investment Fund Foundation (CIFF) (London, UK) and the National Institute for Health and Care Research (NIHR, UK). The studies reviewed include the NICHD-funded Aspirin Supplementation for Pregnancy Indicated Risk Reduction In Nulliparous Women (ASPIRIN) study, the BMGF-funded Project to Understand and Research Pregnancy Outcomes and Stillbirths in South Asia (PURPOSe) study, the NICHD-funded Global Network’s COVID-19 studies, the CIFF-funded Reducing Iron Deficiency Anaemia in Pregnancy in India (RAPIDIRON) study, the NIH–National Cancer Institute (NCI)-funded CryoPop study, the BMGF-funded antenatal corticosteroid (ACS) studies, the Low-birth weight Infant Feeding Exploration Study (LIFE) and the studies from the NICHD-funded Global Network’s Maternal and Newborn Health Registry (MNHR).

Although a large number of topics are addressed in this supplement, it is important to emphasise several recurrent themes. The first is the emphasis on placental studies. Hoffman wrote an article on the ‘Great Obstetrical Syndromes’, which, among other points, emphasised the role of the placenta in the pathway to most of the important pregnancy outcomes.^1^ He emphasised that placental malperfusion lesions, which generally have their origins in the first trimester, are at least partially responsible for stillbirth, preterm birth, pre-eclampsia, placental abruption and fetal growth restriction. This article arose in part from the ASPIRIN trial, which was led by the Research Unit at JNMC.^2^ One of the secondary outcomes of the ASPIRIN trial was the impact of low-dose aspirin (LDA) on hypertensive disorders of pregnancy (HDP). LDA reduced the incidence of preterm HDP but did not show a reduction in the overall incidence of HDP. A post-hoc exploratory analysis of the ASPIRIN trial by Kavi et al. reported that women who received LDA were less likely to have HDP at delivery before 34 and 37 weeks of gestation. This suggested that LDA improves pregnancy outcomes by delaying the onset of HDP, thereby extending the gestational age at delivery.^3^

The JNMC Research Unit has a long-standing interest in understanding the causes of stillbirth and neonatal mortality and ways to prevent these outcomes. The PURPOSe study evaluated causes of stillbirth and preterm neonatal death and confirmed that the majority of stillbirths and about half of preterm neonatal deaths were caused by birth asphyxia, often linked to placental malperfusion.^4^ An evaluation of placental lesions associated with pre-eclampsia confirmed the importance of placental lesions in the impact of pre-eclampsia on pregnancy outcomes.^5^

The PURPOSe study data are unique and informative in determining the cause of deaths among stillbirths and neonatal deaths.^4^ Maternal clinical history, placental histology and fetal examination were most informative. The study adopted minimally invasive tissue sampling (MITS) techniques, replacing conventional autopsy methods, and fetal tissue histology and polymerase chain reaction (PCR) identified many aetiological agents. These novel techniques will ascertain the most common causes of stillbirth and most of the preventable causes of stillbirth in LMICs. This study not only evaluated malperfusion lesions but also evaluated inflammatory lesions. Of the inflammatory lesions, including chorioamnionitis, funisitis, villitis and intervillitis, chorioamnionitis was not only the most common lesion but was also associated with stillbirth, as was funisitis or inflammation of the umbilical cord. *Ureaplasma urealyticum*, the organism most commonly found in the placenta, was associated with various types of inflammation.^6^ The PURPOSe study also used multiplex PCR analyses to evaluate 73 organisms and their presence in various tissues in neonatal deaths.^7^ In the article by Kallapur et al.,^8^ an interesting observation was that Group B *Streptococcus* (GBS) was rarely found in the placenta or in fetal organs in relationship to stillbirth or neonatal death in India and Pakistan. By far the most common organism found in the tissues of neonatal deaths was *Acinetobacter baumannii*. These cases are likely to represent nosocomial infections, acquired by infants from non-sterile surfaces or from equipment in the neonatal intensive care unit (NICU).^4,7^

The PURPOSe study concluded that the majority of stillbirths were preventable with available care.^4,9^ In addition, Goldenberg et al. evaluated the data needed to accurately determine the causes of death in stillbirths.^10^ An article by Saleem et al., comparing neonatal mortality in NICUs in India and Pakistan, presented reasons for the lower NICU neonatal mortality rate in India compared with Pakistan.^11^ Dr Gowder summarised the impact of twin pregnancy on pregnancy outcomes and noted that preterm twins accounted for nearly 20% of the preterm neonatal mortality.^12^

The JNMC Research Unit participated in several studies on the use of ACS to improve newborn outcomes in LMICs.^13^ The use of ACS in women at high risk of preterm birth (≤34 weeks of gestation) is a proven intervention for preventing several preterm morbidities and neonatal mortality, especially in settings where adequate obstetric and neonatal care, including respiratory support, nutrition, thermoregulation and the management of sepsis, can be provided. Compared with the impact on neonates at <34 weeks of gestation, we do not have conclusive evidence on the possible impact of using ACS in the late preterm period in LMICs. A review by Sultana et al. evaluated the available evidence and persisting uncertainties that apply to the use of ACS in all settings, including the optimal ACS regimen and the effect of ACS on longer-term outcomes.^13^

Dr Georgieff, in his review, summarised the relationship between maternal and infant iron status and infant neurodevelopmental outcomes.^14^ Given the high prevalence of iron deficiency among pregnant women, especially in South Asia, increasing our understanding of the relationship of iron deficiency and outcomes and ways to reduce iron deficiency among pregnant women is a high priority of the JNMC Research Unit.^14^

Globally, knowledge regarding early and optimal feeding practices for small, vulnerable infants is limited. The LIFE project performed a complex, mixed-methods research study on infant feeding.^15^ They explored reducing the challenges and the lessons learned. Vernekar et al. described the process used in this study for developing a comprehensive understanding of the health of preterm infants and planning evidence-based, targeted, large-scale interventions for those babies.^15^

In this supplement, there are several other studies emphasising the JNMC Research Unit’s interest in maternal and neonatal nutrition. One such article highlights the use of peer counsellors to support exclusive breast feeding (EBF). In an exploratory qualitative study, Charantimath et al. established that the use of the peer counsellor intervention model could result in an improvement in EBF rates.^16^ In another article, Salam et al. studied the impact of a school-based education programme on knowledge related to iron-deficiency anaemia, and demonstrated that educational interventions for adolescents conducted by teachers in schools are effective in improving awareness and attitudes related to anaemia.^17^

Climate change and the effect of climate on various pregnancy outcomes is becoming a subject of increasing importance to several Global Network sites. In an article focusing on Global Network sites in South Asia, including Bangladesh, India and Pakistan, Shankar et al. evaluated ambient temperatures in relation to a number of pregnancy outcomes.^18^ They found that high ambient temperatures, especially early in pregnancy, were correlated with increased rates of preterm birth and low birthweight. These results confirmed an earlier finding from Pakistan, published by the same group, in which high temperatures in the first trimester were associated with increased rates of fetal growth restriction. Perhaps most importantly, they found that maternal treatment with multi-micronutrients appeared to reduce the risk of fetal growth restriction.^19^

The Global Network’s COVID-19 studies, which prospectively assessed pregnancy outcomes with COVID-19 antibody status at delivery, took place in the eight sites of the NICHD Global Network from 2020 to 2022 and produced some very interesting results.^20^ The increasing COVID-19 infection rate over time in pregnant women was documented by COVID-19 antibody testing at delivery, with the highest rates of antibody positivity found in the Indian sites. Interestingly, in women testing positive for antibodies who had not been vaccinated, i.e. evidence of a prior COVID-19 infection, adverse pregnancy outcomes including stillbirth, neonatal death, preterm birth and low birthweight were not increased.^20,21^ The most important reason for the lack of association was that in this study, most infected women were asymptomatic, and of those who had symptoms, the symptoms were mild in nature. Very few women had severe disease.^20,21^ In a subset of the population studied, women who tested negative for antibodies early in their pregnancy and who later tested positive at delivery had similar results, suggesting that infection prior to pregnancy did not explain these results. Other studies suggesting more serious outcomes associated with COVID-19 exposure during pregnancy were often hospital-based and generally included COVID-positive women who were recruited in hospitals and were often quite ill.^20,21^ The JNMC Research Unit also investigated whether putative COVID-19 symptoms could be used to diagnose COVID-19 infections during pregnancy.^22^ In this analysis conducted by Kavi et al., compared with women who were antibody-negative, women who were antibody-positive had slightly more episodes of fever during pregnancy, but the differences in symptomatology between women who were antibody-positive and women who were antibody-negative were not enough to suggest the use of symptoms as a diagnostic tool for COVID-19 infection.^22^

The Global Network also evaluated the use of COVID-19 vaccinations across the network over time. The use of COVID-19 vaccines in pregnant women at the sites increased dramatically from 2020 through to 2022 and the Indian sites had among the highest rates of vaccination across the Global Network.^20^ The Global Network studied knowledge, attitudes and practices (KAP) related to COVID-19 and COVID-19 vaccinations across sites and time.^23^ We were impressed with the wide variability of COVID-19 knowledge across the sites. The major change in practice was that over time, fewer women planned to avoid healthcare providers through a fear of contacting COVID-19 from their providers.^23^ Pregnant women at the Global Network sites, including those in India, became more accepting of COVID-19 vaccinations over time, mostly as a result of recommendations from healthcare providers and decreasing concern related to vaccine safety.

An article in this supplement on screening and treatment for high-grade cervical dysplasia, at first consideration, might seem like an outlier in this issue with the other articles mostly focusing on pregnancy outcome. However, the expansion of research topics covered by the JNMC Research Unit is an indication that the unit leadership wants to be involved in women’s health research beyond pregnancy.^24^

An important article, the last in this supplement, is titled ‘Connecting the dots: Adoption of maternal, newborn and child health research evidence in policy and practice’ by Chandhiok et al.^25^ This article makes the crucial point that completing a research study – even with positive results that confirm benefits and suggest a change in practice – is often not enough to actually result in a change in practice. The article suggests a number of methodologies to more effectively incorporate research results into practice.^25^

One other observation is important. The JNMC Research Unit has expanded its research to include other universities in India, as illustrated in articles from faculty members at the universities in Davanagere;^8,12^ and Bagalkot.^17^ The partnership between the research teams from KLE Academy of Higher Education and Research and Aga Khan University in the PURPOSe study and the Global Network studies on COVID and climate enable the generalisability of the results to South Asia, and bodes well for further research partnerships.

## References

[1] Hoffman MK. The great obstetrical syndromes and the placenta. BJOG. 2023;130(Suppl 3):8–15. 37530495 10.1111/1471-0528.17613 PMC10834844

[2] Hoffman MK, Goudar SS, Kodkany BK, Metgud M, Somannavar M, Okitawutshu J, Low-dose aspirin for the prevention of preterm delivery in nulliparous women with a singleton pregnancy (ASPIRIN): a randomised, double-blind, placebo-controlled trial. Lancet. 2020;395:285–93. 31982074 10.1016/S0140-6736(19)32973-3 PMC7168353

[3] Kavi A, Hoffman MK, Somannavar MS, Metgud MC, Goudar SS, Moore J, Aspirin delays the onset of hypertensive disorders of pregnancy among nulliparous pregnant women: a secondary analysis of the ASPIRIN trial. BJOG. 2023;130(Suppl 3):16–25. 37470099 10.1111/1471-0528.17607 PMC10799162

[4] Goldenberg RL, Saleem S, Goudar SS, Moore J, Gowdar G, Kulkarni V, The PURPOSe cause of death study in stillbirths and neonatal deaths in India and Pakistan: a review. BJOG. 2023;130(Suppl 3): 26–35. 37592743 10.1111/1471-0528.17635

[5] Yogeshkumar S, Dhananjay S, Gowdar S, Gowdar G, Kulkarni V, Byranahalli S, Morphological study of placenta in deliveries with pregnancy induced hypertension – results from a prospective, observational study (PURPOSe). BJOG. 2023;130(Suppl 3):36–42. 37530629 10.1111/1471-0528.17617

[6] Ahmed I, Ghanchi NK, Tikmani SS, Jackson K, Kim J, Zafar A, Placental inflammation and pregnancy outcomes: a prospective, observational study in South Asia: the PURPOSe study. BJOG. 2023;130(Suppl 3):43–52. 37671586 10.1111/1471-0528.17643

[7] Ghanchi NK, Ahmed I, Kim J, Harakuni S, Somannavar MS, Zafar A, Pathogens identified by minimally invasive tissue sampling in India and Pakistan from preterm neonatal deaths: the PURPOSE study. Clin Infect Dis. 2023;76(3):e1004–11. 36104850 10.1093/cid/ciac747 PMC9907547

[8] Kallapur M, Ghanchi NK, Harakuni SU, Somannavar MS, Ahmed I, Fogleman E, Group B streptococcal prevalence in internal organs and placentas of deceased neonates and stillbirths in South Asia. BJOG. 2023;130(Suppl 3):53–60. 37530593 10.1111/1471-0528.17614

[9] Goldenberg RL, Saleem S, Goudar SS, Silver RM, Tikmani SS, Guruprasad G, Preventable stillbirths in India and Pakistan: a prospective, observational study. BJOG. 2021;128(11):1762–73. 34173998 10.1111/1471-0528.16820

[10] Goldenberg RL, Hwang K, Saleem S, Tikmani SS, Yogeshkumar S, Kulkarni V, Data useful in determining cause of stillbirth in South Asia (PURPOSe). BJOG. 2023;130(Suppl 3):61–67. 37470078 10.1111/1471-0528.17592

[11] Saleem S, Tikmani SS, Goudar SS, Hwang K, Dhaded SM, Gowdar G, Neonatal mortality among preterm infants admitted to neonatal intensive care units in India and Pakistan: a prospective study (PURPOSe). BJOG. 2023;130(Suppl 3):68–75. 10.1111/1471-0528.17581 37470084

[12] Gowdar G, Raghoji CR, Dhaded SM, Tikmani SS, Saleem S, Goudar SS, Pregnancy outcomes in preterm multiple gestations: results from a prospective study in India and Pakistan (PURPOSe). BJOG. 2023;130(Suppl 3):76–83. 37470087 10.1111/1471-0528.17602

[13] Sultana S, Vogel JP, Oladapo OT. The efficacy of antenatal corticosteroids to improve preterm newborn outcomes in low-resource countries: are we there yet? BJOG. 2023;130(Suppl 3):84–91. 37530472 10.1111/1471-0528.17611

[14] Georgieff M Maternal gestational iron status and infant hematologic and neurodevelopmental outcomes. BJOG. 2023;130(Suppl 3):92–98. 37530464 10.1111/1471-0528.17612

[15] Vernekar SS, Somji S, Msimuko K, Yogeshkumar S, Nayak RB, Nabapure S, Lessons learned in implementing the Low Birthweight Infant Feeding Exploration (LIFE) study: a large, multi-site observational study. BJOG. 2023;130(Suppl 3):99–106. 10.1111/1471-0528.17603 37470090

[16] Charantimath US, Bellad RM, Mahantashetti NS, Karadiguddi CC, Mungarwadi GM, Kelly PJ, Experiences of peer counsellors in supporting exclusive breastfeeding in southern India: an exploratory qualitative study. BJOG. 2023;130(Suppl 3):107–112. 10.1111/1471-0528.17615 PMC13281724 37530600

[17] Salam SS, Ramadurg UY, Charantimath US, Katageri GM, Gillespie B, Mhetri J, Impact of a school-based nutrition educational intervention on knowledge related to iron deficiency anaemia in rural Karnataka, India: a mixed methods pre-post interventional study. BJOG. 2023;130(Suppl 3):113–123. 37530624 10.1111/1471-0528.17619

[18] Shankar K, Jackson K, Westcott JL, Saleem SS, Aziz SA, Jessani S, Associations between ambient temperature and pregnancy outcomes from three South Asian sites of the Global Network Maternal Newborn Health Registry: a retrospective cohort study. BJOG. 2023;130(Suppl 3):124–133. 37581948 10.1111/1471-0528.17616 PMC10843605

[19] Shankar K, Ali SA, Ruebel ML, Jessani S, Borengasser SJ, Gilley SP, Maternal nutritional status modifies heat-associated growth restriction in women with chronic malnutrition. PNAS Nexus. 2023;2(1):pgac309. 10.1093/pnasnexus/pgac309 36744021 PMC9896899

[20] Naqvi S, Saleem S, Billah SKM, Moore JL, Mwenechanya M, Carlo WA, The Global Network COVID-19 studies: a review. BJOG. 2023;130(Suppl 3):134–139. 37530467 10.1111/1471-0528.17610 PMC11256983

[21] Goldenberg RL, Saleem S, Billah SM, Kim J, Moore JL, Ghanchi NK, COVID-19 antibody positivity over time and pregnancy outcomes in seven low-and-middle-income countries: a prospective, observational study of the Global Network for Women’s and Children’s Health Research. BJOG. 2023;130(4):366–76. 36504437 10.1111/1471-0528.17366 PMC9877904

[22] Kavi A, Goudar SS, Somannavar MS, Moore JL, Derman RJ, Saleem S, COVID-19 symptoms and antibody positivity among unvaccinated pregnant women: an observational study in seven countries from the Global Network. BJOG. 2023;130(Suppl 3):140–148. 37470094 10.1111/1471-0528.17604 PMC10799161

[23] Jessani S, Saleem S, Fogleman E, Billah SKM, Haque R, Figueroa L, Trends over time in pregnant women’s knowledge, attitude and practices related to COVID-19: a cross-sectional survey from 7 low-and middle-income countries. BJOG. 2023;130(Suppl 3):149–157. 37581947 10.1111/1471-0528.17630 PMC11259376

[24] Anderson J, Yogeshkumar S, Lu E, Yenokyan G, Thaler K, Mensa M, The CryoPop study: screening for high grade cervical dysplasia in Karnataka, India: a prospective cohort study. BJOG. 2023;130(Suppl 3):158–167. 37932903 10.1111/1471-0528.17702 PMC10659137

[25] Chandhiok N, Goudar SS, Kavi A, Somannavar MS, Silver RM. Connecting the dots: adoption of maternal, newborn and child health research evidence in policy and practice. BJOG. 2023;130(Suppl 3):168–171. 37530407 10.1111/1471-0528.17599

